# Calculation of the Aqueous Thermodynamic Properties of Citric Acid Cycle Intermediates and Precursors and the Estimation of High Temperature and Pressure Equation of State Parameters

**DOI:** 10.3390/ijms10062809

**Published:** 2009-06-22

**Authors:** Peter Dalla-Betta, Mitchell Schulte

**Affiliations:** 1 School of Earth and Space Exploration, Arizona State University, Box 871404, Tempe, AZ 85287, USA; Current address: RCM Digesters, Inc., P.O. Box 4716, Berkeley, CA 94704, USA; E-Mail: pete.dalla-betta@earthlink.net (P.D.-B.); 2 Laboratory Exploring Biogeochemistry and Astrobiology Research, Department of Geological Sciences, University of Missouri, 101 Geology Building, Columbia, MO 65211, USA

**Keywords:** citric acid cycle, thermodynamics, metabolism

## Abstract

The citric acid cycle (CAC) is the central pathway of energy transfer for many organisms, and understanding the origin of this pathway may provide insight into the origins of metabolism. In order to assess the thermodynamics of this key pathway for microorganisms that inhabit a wide variety of environments, especially those found in high temperature environments, we have calculated the properties and parameters for the revised Helgeson-Kirkham-Flowers equation of state for the major components of the CAC. While a significant amount of data is not available for many of the constituents of this fundamental pathway, methods exist that allow estimation of these missing data.

## 1. Introduction

The citric acid cycle (CAC), also known as the Krebs or tricarboxylic acid cycle, is a fundamental pathway in intermediary metabolism among all the domains of life. Organisms that use the CAC (or especially the reverse or reductive CAC) are represented among the most deeply-rooted autotrophic hyperthermophilic Archaea and Bacteria and the most derived of organotrophic evolutionary lineages [1–4]. The key role of iron-sulfur proteins, thioester intermediates, and the reductive use of the CAC in hyperthermophilic autotrophs link the CAC to prebiotic theories of energy metabolism and abiotic carbon fixation at deep-sea hydrothermal vents [5–7]. Because of the fundamental metabolic roles and evolutionary importance of the CAC and the reverse CAC, it is of interest to understand the conditions constraining the many reactions of which it may be composed under the considerable range of physical and chemical environments where it functions. Most thermodynamic data available for the substrates used in the CAC are incomplete and have only been determined at the 25 °C and 0.1 MPa standard state. Therefore it is difficult to predict accurate reaction thermodynamics well beyond those conditions, including those likely to have hosted the emergence of life [7–10]. For examination of CAC reactions under the relatively extreme high-temperature and high-pressure conditions where life can thrive and may have originated, and to determine the geochemical environments where prebiotic conditions may have been favorable, the high pressure and temperature thermodynamic parameters of the substrates must be determined.

To evaluate the standard Gibbs free energy ( $\Delta G_{r}^{0}$) of reaction for a given set of products and reactants at temperatures and pressures beyond the 25 °C – 0.1 MPa reference conditions in an aqueous system, the standard Gibbs free energy of formation ( $\Delta G_{f}^{0}\;aq$) for each substance at non-standard temperature and pressure must be calculated. Most thermodynamic data available for aqueous organic molecules beyond the 25 °C and 0.1 MPa reference conditions are generally for compounds that are either potential growth substrates or metabolic by-products. Only in a few works are there data available for compounds that are intermediates in the ubiquitous biochemical pathways found in nature. The general lack of empirical data for aqueous organic compounds at elevated temperature and pressure has lead to the development of various equations of state to describe the behavior of various thermodynamic properties at non-standard temperature and pressure [11,12]. The revised Helgeson-Kirkham-Flowers equations of state [11,13], along with methods for the estimation of high pressure and temperature thermodynamic properties [14–20], can be used to predict accurately reaction thermochemical properties for aqueous organic compounds well within the thermal and pressure range of the possible environments where these reactions may be expected to occur in biological processes. In addition to aqueous organic compounds [13,18,21,22], the revised HKF equations of state have been used to calculate accurately the thermodynamic reaction properties, without the benefit of high pressure and temperature data, for aqueous inorganic [17] and organic ions [13,23], and inorganic electrolytes [19] at subcritical temperatures and pressures up to 500 MPa. Such versatility makes it one of the more useful tools for evaluating geochemical and biochemical reaction properties in a wide variety of environments, as the revised HKF allows calculation of species and reaction properties among minerals, gases and aqueous species. This provides a framework for understanding many interdependent metabolic and geochemical processes [24].

**Figure 1.**
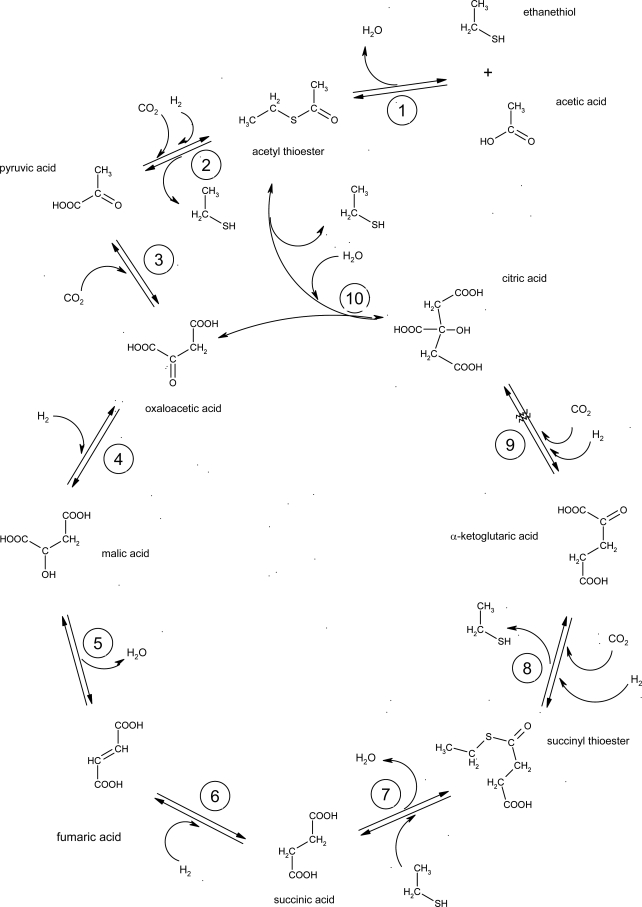
The reverse citric acid cycle (modified from [25]).

Data for reactions depicted in Figure 1 are used as a suggestion for a prebiotic reductive CAC. However, estimation of the thermochemical properties of the thioester intermediates may also function as proxies in lieu of high pressure and temperature reaction data for coenzyme A or other thioester intermediates found in Archaea.

## 2. Methods

The revised HKF equation of state uses the standard [26] $\Delta G_{f}^{0}\;aq$, aqueous partial molal entropy (*S*^0^*aq*), partial molal volume (*V*^0^), and constant pressure molal heat capacity ( $C_{P}^{0}$), along with fitting parameters that integrate the change in the partial molal property, into the $\Delta G_{f}^{0}\;aq$ at the desired temperature and pressure conditions. In regard to the revised- HKF equation of state, the 25 °C – 0.1 MPa properties are referred to as the reference state, i.e. $\Delta G_{f}^{0}\;aq_{P_{r},T_{r}}$, and the calculated partial molal property at P and T, the standard state, i.e. $\Delta G_{f}^{0}\;aq_{P,T}$. For substances for which incomplete thermodynamic data are available, the use of group contribution, or additivity, algorithms has become the most pragmatic method available in light of the vast number of naturally occurring and synthetic organic compounds. Group additivity relationships have been used to generate 25 °C, 0.1 MPa reference state thermodynamic properties and group values. These estimation methods have been used for pure phase gas, liquid and solid organic compounds [27–29] and aqueous neutral and ionic organic compounds. Namely among the aqueous species are *n*-alkanes, *n*-alkenes, *n*-alcohols, *n*–alkanones [30], aldehydes [15], amino acids [18,22], carboxylic, hydroxy and dicarboxylic acids, and their respective ions [23], as well as numerous biochemically-relevant organic compounds (e.g.[31,32]).

### 2.1. Strategy used for the estimation of missing reference state values

**Figure 2.**
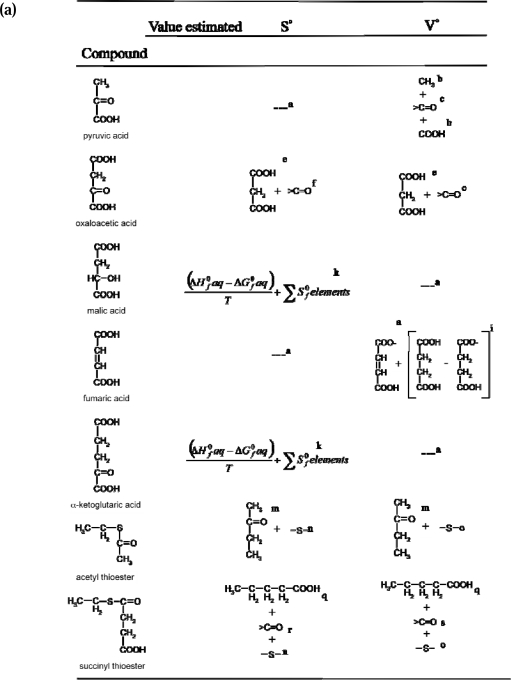

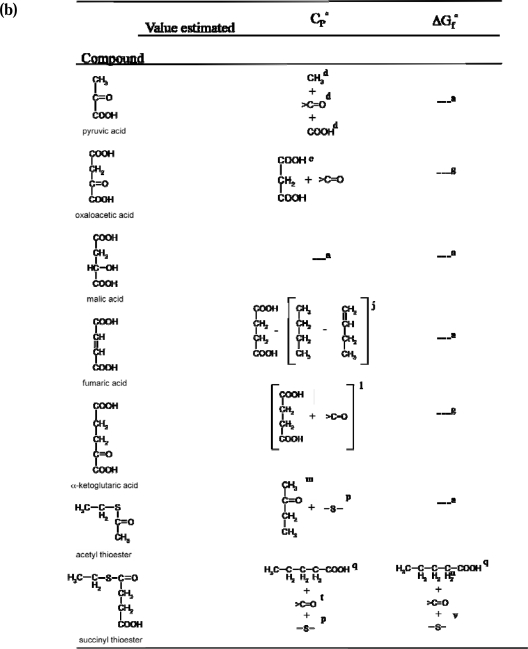
Group contribution method used to estimate (a) the standard partial molal entropy (S^°^) and volume (V^°^) and (b) the heat capacity (C_p_°) and free energy (ΔG_f_°) of aqueous organic species considered in this study. ^a^-value taken from reference shown in Table 1. ^b^- group values from [35]. ^c^- the carbonyl group value was estimated from the difference in *V*^0^ between succinic acid [23] and α-ketoglutarate [36]. ^d^-estimated using group values from [31]. ^e^-malonic acid from [23]. ^f^-group $S_{f}^{0}aq$ value for >C=O assumed to be the difference in $S_{f}^{0}aq$ between α-ketoglutaric acid [37] and succinic acid [23]. ^g^-calculated form *p*Ka values from [33] and $\Delta G_{f}^{0}aq$ for ions from Table 1 (as described in text). ^h^-value for $\Delta G_{f}^{0}aq$ was taken from [38], the $\Delta H_{f}^{0}aq$ was from [33], and $S_{f}^{0}\text{elements}$ were the CODATA values from [39]. ^i^-estimated from *V*^0^ of H-fumarate^−1^ (Figure 2) assuming the same difference in *V*^0^ between the acid and ion as between succinic acid and H-succinate^−1^ from [23]. ^j^-estimated by subtracting the difference in y-intercept values for $C_{P}^{0}$ between *n*-alkanes and *n*-alkenes (taken from [18]) from that of succinic acid (taken from [23]). ^k^- calculated from *p*Ka and $\Delta G_{f}^{0}aq$ values from [33] for ions (as described in text), $\Delta H_{f}^{0}aq$ was from [37)] and $S_{f}^{0}\text{elements}$ were the CODATA values from [39]. ^l^-the $C_{P}^{0}$ for succinic acid was taken from [23]. The group value for the >C=O was taken from [31]. ^m^-from [18]. ^n^-group value estimated from difference in $S_{f}^{0}aq$ values between ethyl sulfide (as described in Appendix) and *n*-butane (taken from [18]). ^o^- group value from [40]. ^p^- group value estimated from difference in $C_{P}^{0}$ values between ethyl sulfide [34] and *n*-butane (taken from [18]. ^q^-from [23]. ^r^-group value estimated from difference in y-intercepts of $S_{f}^{0}aq$ between *n*-alkanones and n-1 *n*-alkanes taken from [18]. ^s^- group value from [30]. ^t^- group value estimated from difference in y-intercepts of $C_{P}^{0}$ between *n*-alkanones and n-1 *n*-alkanes taken from [18]. ^u^- group value estimated from difference in y-intercepts of $\Delta G_{f}^{0}aq$ between *n*-alkanones and n-1 *n*-alkanes taken from [18]. ^v^- group value estimated from difference in $\Delta G_{f}^{0}aq$ values between ethyl sulfide (calculated from $\Delta G_{f}^{0}g$ and Δ*_hyd_G* from [34] and *n*-butane (taken from [18)].

The approach taken to estimate reference state values herein is to utilize methods using state variables and reaction data, where available, to calculate missing values. In absence of state or reaction data, the group contribution method is used, with emphasis on attaining data for the closest structural analogues as a base structure. In doing so, the fewest group values are used to modify the base structure as a means of decreasing the probability of error accumulation. The selection of methods used to estimate reference state data are summarized in Figure 2 using the neutral species as examples.

### 2.2. Calculation of reference state $\Delta G_{f}^{0}\;aq$ and estimation of missing values

The $\Delta G_{f}^{0}\;aq$ values selected for neutral and ionic organic species are shown in Table 1. The $\Delta G_{f}^{0}\;aq$ for organic acids and ions for which there were no data available were calculated from the ionization constants (*p*Ka), critically compiled in [33], and $\Delta G_{f}^{0}\;aq$ of the respective ion or acid for which there were reliable data. The only acid-anion pair for which $\Delta G_{f}^{0}\;aq$ values were unavailable was succinyl thioester. Therefore, estimation of its $\Delta G_{f}^{0}\;aq$ was necessary and attained through the addition of the $\Delta G_{f}^{0}\;aq$ for the (>C=O) and (–S-) groups to pentanoic acid-pentanoate $\Delta G_{f}^{0}\;aq$ for the succinyl thioester acid-anion pair. The $\Delta G_{f}^{0}\;aq\;(>\text{C}=\text{O})$ group value was calculated from the difference in the y-intercepts for regression equations between *n-*alkanones and (n-1) *n*-alkanes (both taken from reference [18]). The $\Delta G_{f}^{0}\;aq\;(-\text{S}-)$ was calculated similarly, from the difference in y-intercepts from dialkylsulfides [34] and *n*-alkanes [18], with the same number of carbon atoms. Data for other components of the citric acid cycle (ethyl thiol and acetic acid; see Figure 1) are available in the literature [16,23].

The estimation of high temperature and pressure thermochemical properties for *cis*-aconitate and isocitrate, for the reactions between citrate and α-ketoglutarate, were not calculated due to deficient reference state data. Since citric acid and its ions are the only feasible tricarboxylated base structures available to use for the estimation, this approach was rejected to avoid circularity. Furthermore, the primary goal of estimating the high pressure and temperature parameters for CAC reactions calculated herein is primarily driven to examine these reactions under the near equilibrium conditions that are present in anoxic systems. Extant microbes that use the CAC reductively to fix carbon generally only contain a partial cycle where the pathway is used to supply precursors for biosynthesis via succinyl-Coenzyme A or α-ketoglutarate [2–4,45].

### 2.3. *Calculation of the reference state entropy (S*^0^ *aq) and estimation of missing values*

The *S*^0^*aq* values selected are shown in Table 1. If *S*^0^*aq* values were not available, third-law entropies were calculated from the aqueous enthalpy and Gibbs free energy values along with the sum of entropies of the elemental constituents using Equation (1):

(1) $$S^{0}aq=\frac{\Delta H_{f}^{0}aq-\Delta G_{f}^{0}aq}{T}-\sum S_{\text{elements}}^{0}$$

The *S*^0^*aq* for some anions in Table 1 were calculated from the *S*^0^*aq* of the acid using Δ*_ion_S* for the ionization reactions from Miller and Smith-Magowan [33]:

(2) $$S^{0}aq\;(\text{acid})=S^{0}aq\;({\text{ion}}^{-1})+\Delta_{ion}S$$

The aqueous formation entropies for H-α-ketoglutarte^−1^ and α-ketoglutarte^−2^ were estimated by assuming similar Δ*_ion_S* from succinic acid and its mono- and divalent anions (from Shock [23]). The carbonyl group *S*^0^*aq* contribution value (54.3 J mol^−1^) used for oxaloacetic acid and its respective anions was calculated from the difference in *S*^0^*aq* between α-ketoglutaric acid and succinic acid from Shock [23]:

(3) $$S^{0}aq\;(\alpha -\text{ketoglutaric}\;\text{acid})\;-\;S^{0}aq\;(\text{succinic}\;\text{acid})=S^{0}aq\;(>\text{C}=\text{O})$$

To calculate the *S*^0^*aq* needed for acetyl thioester the value of *S*^0^*aq* (-S-) was calculated from the difference in aqueous entropies between ethyl sulfide (see Appendix A, Equation 21), and *n*-butane [18].

The *S*^0^*aq* (>C=O) value used for succinyl thioester and its anion was estimated from the difference (68.15 J mol^−1^ K^−1^) in y-intercepts between *n*-alkanones and (n-1) *n*-alkanes, both from Shock and Helgeson [18]. The *S*^0^*aq* (>C=O) value used here was chosen since the value derived from an *n*-alkanone may be more likely to represent the value of a subterminal carbonyl adjacent to a sulfur atom, as opposed to the α-carbonyl adjacent to a carboxylic acid, as was used above for oxaloacetic acid.

### 2.4. Calculation of the reference state partial molal volume (V°aq) and estimation of missing values

The chosen partial molal volume values are shown in Table 1. The group value *V*^0^ (>C=O) used for the oxaloacetate series was estimated by the difference in *V*^0^ between α-ketoglutaric acid [36] and succinic acid [35] such that:

(4) $$V^{0}\;(\alpha -\text{ketoglutaric}\;\text{acid})\;-\;V^{0}\;(\text{succinic}\;\text{acid})=V^{0}\;(>\text{C}=\text{O}).$$

This *V*^0^ (>C=O) was used for pyruvic acid and pyruvate as well since it may rather well represent the volume of an α-carbonyl (*V*^0^ ~5 cm^3^ mol^−1^) adjacent to a carboxylic acid, rather than *n*-alkanone’s carbonyl with a much larger volume of (~14–15 cm^3^ mol^−1^ [30,31]).

The *V*^0^ for fumarate^−2^ was used to estimate the *V*^0^ of fumaric acid and the H-fumarate^−1^ anion by assuming the same Δ*_ion_V* relationship for fumaric acid and its ions as for succinic acid from Criss and Wood [35], and H-succinate^−1^ or succinate^−2^ anion, from Shock [23], as demonstrated in Figure 2. The same relationship, using the Δ*_ion_V* (13.13 cm^3^ mol^−1^) between lactic acid and lactate from Shock [23] was used to estimate the *V*^0^ for pyruvate from its acid.

The partial molal volume of the (-S-) group for a thioester was estimated by the addition of the value of *V*^0^ _(-S-)_ from a value published by Lepori and Gianni [40] to the *V*^0^ of 2-butanone taken from Shock and Helgeson [18]. The *V*^0^ of succinyl thioester and its anion were estimated by the addition of *V*^0^ _(-S-)_ [40] and *V*^0^ _(>C=O)_ to the *V*^0^ of pentanoic acid and pentanoate, respectively (both from Shock [23]). The *V*^0^ _(>C=O)_ value was estimated to be the difference between the *V*^0^ of *n*-alkanones and (n-1) *n*-alkanes (both taken from Shock and Helgeson [18]).

### 2.5. Calculation of the reference state heat capacity (C°_p_ aq) and estimation of missing values

The standard molal isobaric heat capacities at the reference temperature and pressure are shown in Table 1 and estimation procedures in Figure 2. The $C_{P}^{0}$ of pyruvate ion was assumed to have the same difference in $\Delta_{ion}C_{P}^{0}$ from its acid, as did the lactic acid-lactate pair [23], of 167.4 J mol^−1^ K^−1^. The $C_{P}^{0}$ for oxaloacetic acid was estimated by adding the $C_{P}^{0}\;(>\text{C}=\text{O})$ value (−52.0 J mol^−1^ K^−1^) from that reported by Cabani *et al*. [31] to that of malonic acid [23]. For the estimation of $C_{P}^{0}$ values for both H-oxaloacetate^−1^ and oxaloacetate^−2^ ions, it was assumed that the difference from the acid was the same as between succinic acid and it respective anions from Shock [23]. This assumption was also used to estimate $C_{P}^{0}$ values of the H-mono- and divalent anions of malate, fumarate, and α-ketoglutarate. The $C_{P}^{0}$ of fumaric acid was estimated through subtracting the values for the difference (70.7 J mol^−1^ K^−1^) in y-intercepts between *n*-alkanes from *n*-alkenes (both values being from Shock and Helgeson [18]) from that of succinic acid. For α-ketoglutaric acid, the $C_{P}^{0}\;(>\text{C}=\text{O})$ value (−52.0 J mol^−1^ K^−1^) reported by Cabani *et al*. [31] was added to that of succninc acid. The $C_{P}^{0}$ for succinyl thioester was estimated from addition of the $C_{P}^{0}\;(>\text{C}=\text{O})$, and the $C_{P}^{0}\;(-\text{S}-)$ (−81.2 J mol^−1^ K^−1^), both calculated in this work, to the $C_{P}^{0}$ of pentanoic acid from Shock [23]. The $C_{P}^{0}\;(-\text{S}-)$ for succinyl thioester was calculated from the difference in y-intercepts of C_2_,C_4_,C_6_ n-diaklysulfides [34] and C_2_,C_4_,C_6_ n-alkanes [23]. The $C_{P}^{0}\;(>\text{C}=\text{O})$ value (−135.2 J mol^−1^ K^−1^) was calculated to be the difference between 2-pentanone and *n*-butane, both from Shock and Helgeson [18]. This value for $C_{P}^{0}\;(>\text{C}=\text{O})$ was chosen for succinyl thioester because a carbonyl from an *n*-alkanone may be more likely to represent the $C_{P}^{0}\;(>\text{C}=\text{O})$ in a thioester, as opposed to that from a α-carbonyl adjacent to a carboxylic acid. The resulting value of 216.1 J mol^−1^ K^−1^ is in close agreement with the sum of group values provided by Cabani *et al*. [31], using the (-S-) calculated here, of 216.4 J mol^−1^ K^−1^. The $C_{P}^{0}$ of the succinyl thioester anion was assumed to have the same difference from its acid as that between pentanoic acid and pentanoate [23]. The $C_{P}^{0}$ of acetyl thioester was estimated by adddition $C_{P}^{0}\;(-\text{S}-)$, as described above, to that of 2-butanone from Shock and Helgeson [18].

### 2.6. Extrapolation of reference state data to high pressures and temperatures

Starting with 25°C, 0.1 MPa reference state data, calculating the $\Delta G_{f}^{0}aq_{P,T}$ at the elevated P and T involves the integration of Equation (5) as:

(5) $$\Delta G_{P,T}^{0}=\Delta G_{P_{r},T_{r}}^{0}-S_{P_{r},T_{r}}^{0}(T-T_{r})+\;\int_{T_{r}}^{T}C_{P}^{0}dT-T\int_{T_{r}}^{T}C_{P}^{0}d\;\text{ln}\;T+\int_{P_{r}}^{P}V^{0}\;dP$$

The revised-HKF model allows for the incorporation of the changes in the partial molal volume and heat capacity as described in Appendix B.

### 2.7. Estimation of the temperature and pressure effects on the partial molal volume of aqueous organic species: the non-solvation contribution

**Figure 3.**
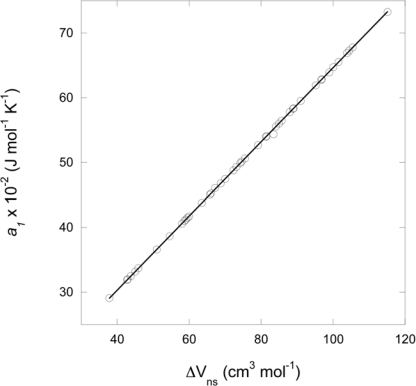
Regression plot of the non-solvation parameter *a_1_* against the non-solvation volumes of short-chained aqueous organic species taken from the literature [15,18,23]. The non-solvation volumes were calculated using Equation (25) with the partial molal volumes and effective Born coefficients of (C_3_-C_5_) carboxylic acids, (C_2_-C_6_) carboxylate anions, (C_3_-C_5_) hydroxy acids, (C_3_-C_6_) hydroxylate anions, (C_2_-C_6_) dicarboxy acids, dicarboxylate^−1^, and dicarboxylate^−2^ anions [23], (C_3_-C_5_) *n*-alkanones, *n*-alkanes, *n*-alkenes, *n*-alcohols [18] and (C_3_-C_5_) aldehydes [15].

The partial molal volume of a substance in the revised HKF model is defined by Equation (24). At temperatures of ≤ ~150°C, at the water-saturation vapor pressure (P_sat_), the solute-dependent contribution (the non-solvation volume, $\Delta V_{n}^{0}$) to the partial molal volume term dominates the partial molal volume. The $\Delta V_{n}^{0}$ term is calculated from Equation (25) utilizing fitting parameters (*a*_1_, *a*_2_, *a*_3_, and *a*_4_) to integrate the $\Delta V_{n}^{0}$ term into *V*^0^ at a desired pressure and temperature. The *a*_1_ and *a_2_* parameters have been generated from empirical data gathered at high pressure and temperature conditions for a variety of compounds [11,15–20,46,47]. The *a*_1_ variable is correlated to a high degree with the $\Delta V_{n}^{0}$ of a wide range of neutral and charged aqueous organic compounds with a variety of functional groups (Figure 3) and can therefore be used to estimate the values of *a*_1_ for compounds with structural homology to those with high pressure and temperature data for which there are no volumetric data beyond the reference state.

For the range of compounds regressed in Figure 3, the line is defined by:

(6) $$a_{1}=0.5711\;\;\Delta V_{n}^{0}+7.4803$$

where *a*_1_ is in J mol^−1^ K^−1^ and $\Delta V_{n}^{0}$ in cm^3^ mol^−1^. This regression equation was used to generate the *a*_1_ parameter for all neutral and charged compounds in this work.

**Figure 4.**
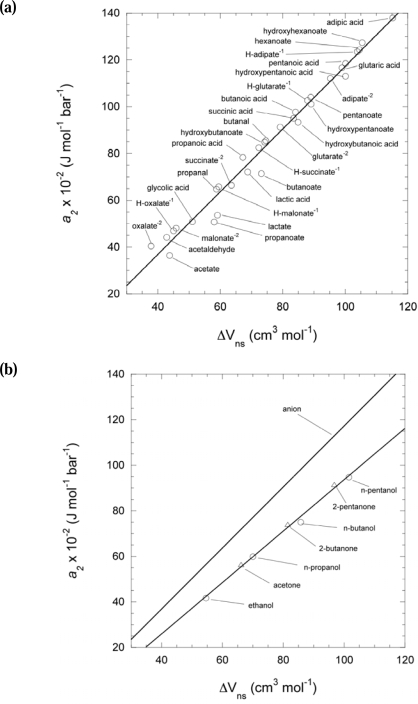
Regression plot of the non-solvation parameter *a_2_* against the non-solvation volumes of short-chained aqueous organic species taken from the literature [15,18,23]. (a) Regression of non-solvation volumes calculated with Equation (25) using the partial molal volumes and effective Born coefficients of (C_3_–C_5_) carboxylic acids, (C_2_–C_6_) carboxylate anions, (C_3_–C_5_) hydroxy acids, (C_3_–C_6_) hydroxylate anions, (C_2_–C_6_) dicarboxy acids, −1, and −2 anions [23], and aldehydes [15]. (b) Upper line: Regression plot generated from points in upper figure. Lower line: Regression of non-solvation volumes calculated with Equation (25) using the partial molal volumes and effective Born coefficients of (C_3_–C_5_) *n*-alkanones, and (C_2_–C_5_) *n*-alcohols [23].

The *a*_2_ parameter is somewhat more dependent on the functional group characteristics of a particular molecule. In Figure 4 the $\Delta V_{n}^{0}$ of aldehydes, hydroxy acids, carboxylic acids, and dicarboxylic acids, and their respective anions, are plotted against *a*_2_.

The data are fit by the line

(7) $$a_{2}=1.341\;\;\Delta V_{n}^{0}\;-\;16.764$$

where *a*_2_ is also in J mol^−1^ K^−1^. For *n*-alkanones and *n*-alcohols (Figure 4) the slope is somewhat shallower and the y-intercept lower so these values are fit better by the line

(8) $$a_{2}=1.129\;\;\Delta V_{n}^{0}\;-\;19.213$$

The *a*_2_ parameter for all dicarboxylic acids, and dicarboxylate anions, citric acid and its anions, pyruvic acid and pyruvate, and succinyl thioester and its anion were estimated with Equation (7). Since the only apparently large departures from the line in Equation (7) are for shorter- chained carboxylate and hydroxylate anions, a fit for hydroxylates was considered separately to calculate the *a*_2_ parameter for pyruvate. The y-intercept for hydroxylates (Equation (9)) is slightly lower with a steeper slope than Equation (8):

(9) $$a_{2}=1.398\;\;\Delta V_{n}^{0}\;-\;14.611$$

However, if the y-intercept value for *n*-alcohols vs. *n*-alkanones can be considered comparable to that of the hydroxy and carbonyl groups in hydroxy- and α keto-acids, respectively, then pyruvate’s y-intercept would shift toward the line in Equation (7). Therefore the *a*_2_ value for pyruvic acid using Equation (7) was retained. The *a*_2_ parameter for acetyl thioester was estimated with the line defined by Equation (8) for *n*-alcohols and *n*-alkanones.

All *a*_4_ parameters were generated, as suggested by Shock and Helgeson and Shock [18,23], using the correlation with the *a*_2_ fitting parameter. The *a*_3_ parameter was then calculated by solving the rearranged non-solvation molal volume term ( $\Delta V_{n}^{0}$) of Equation (25) at 25 °C and 0.1 MPa.

### 2.8. Estimation of temperature and pressure effects on the isobaric heat capacity of aqueous organic species: The non-solvation contribution

**Figure 5.**
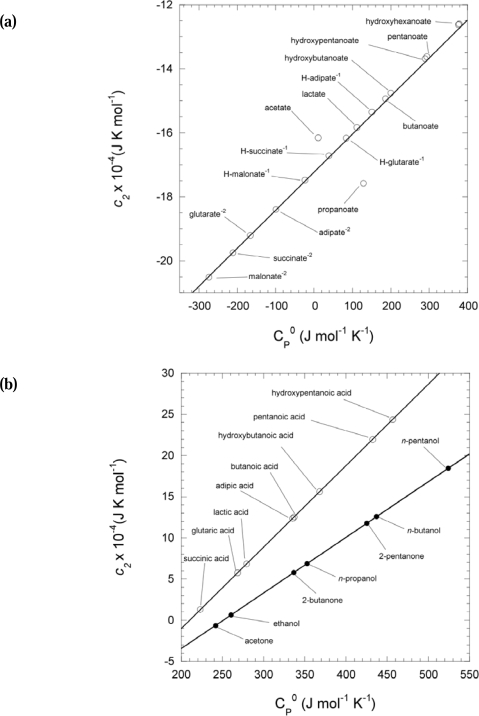
Regression plots of the non-solvation molal heat capacity variable c_2_ against the reference state heat capacity of neutral and ionic organic species from the literature [18,23]. (a) Plot of organic acid anions: (C_2_–C_5_) hydroxylates, (C_2_–C_5_) carboxylates and (C_3_–C_6_) H-dicarboxylate^−1^ and dicarboxylate^−2^ ions (all from reference [23]) used to generate Equation (10). (b) Upper line: Plot of neutral acids: (C_4_–C_5_) carboxylic acids, (C_3_–C_5_) hydroxy acids, and (C_4_–C_6_) dicarboxylic acids from reference [23] used to generate Equation (11). Lower line: Plot of (C_3_–C_5_) *n*-alkanones and (C_2_–C_5_) *n*-alcohols from reference [18] used to generate Equation (12).

The non-solvation and solvation heat capacity contributions to the partial molal heat capacity are defined in Equation (27). The non-solvation contribution term of Equation (27) is expanded in Equation (28) and combines the influence of pressure on the $\Delta C_{P,n}^{0}$ with incorporation of a substance’s *a_3_* and *a_4_* fitting parameters, from above, and the influence of temperature by the use of two heat capacity fitting parameters, *c_1_* and *c_2_*. The *c_2_* parameter correlates closely with the reference state $C_{P}^{0}$ for compounds with similar functional groups (Figure 5).

Therefore, from this correlation high temperature and pressure data from structurally similar compounds can be used to predict the *c_2_* parameter of molecules for which no high temperature heat capacity data are available. Figure 5a shows the plot of *c*_2_ vs. $C_{P}^{0}$ values, taken from Shock [23], for organic acid anions. The regression of these values for the range of compounds shown is described by the line in Equation (10):

(10) $$c_{2}=0.0507\;C_{P}^{0}\;-\;17.188$$

where *c*_2_ is in J mol^−1^. This regression equation was used to estimate the *c*_2_ parameters for all the dicarboxylate and carboxylate anions from the reference state $C_{P}^{0}$ used in this study. Equation (11) describes the upper line in the plot of *c*_2_ vs. $C_{P}^{0}$ values from organic acids (taken from Shock [23]) in Figure 5b:

(11) $$c_{2}=0.4641\;C_{P}^{0}\;-\;25.704$$

which was used to estimate the *c*_2_ parameter for all organic acids. The lower line in Figure 5(b) is a plot of the *c*_2_ vs. $C_{P}^{0}$ of short-chained *n*-alcohols and *n*-alkanones [18] and is described by the line in Equation (12), which was used to estimate the *c*_2_ parameter for acetyl thioester:

(12) $$c_{2}=0.283\;C_{P}^{0}\;-\;16.970$$

### 2.9. Estimation of temperature and pressure effects on the partial molal properties of aqueous organic species: The solvation contribution

At temperatures of ≥~150^°^C, at the water-saturation vapor pressure (P_sat_), the solvent-dependent contribution to the partial molal volume (Equation (26)) and heat capacity (Equation (29)) terms begin to dominate each partial molal function. The conventional (ω) and effective (ω_e_) Born coefficients, respectively, are used to describe the substance-specific solvation properties of an ionic species or electrolytes, and neutral species (see Appendix B).

### 2.10. Calculation of the conventional Born coefficient (ω) for ionic species

**Figure 6.**
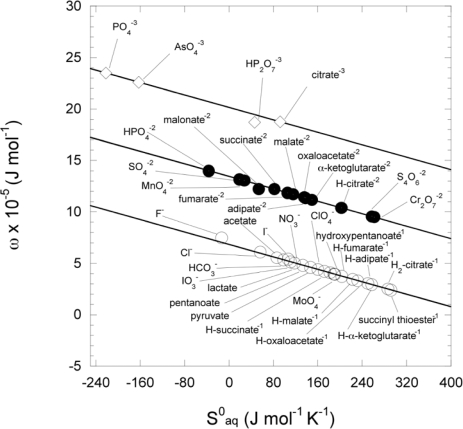
Regression plot of the conventional Born coefficients against the partial molal entropy of various anions. The upper, middle, and lower lines are the correlations for the tri-, di-, and monovalent anions, respectively, from Equations (30–32) using values of inorganic ions (taken from Shock and Helgeson [17]), organic anions (taken from Shock [23]) and compounds calculated in this work.

The ω of ionic species has been demonstrated to have a strong correlation with the $\Delta S_{f}^{0}aq$ [17] and can therefore be calculated from this quantity. In Figure 6 ω is plotted against the $\Delta S_{f}^{0}aq$ for a variety of mono-, di- and trivalent inorganic and organic anions (taken from Shock and Helgeson (1988) [17]) using relationships in Equations (30–32) to calculate ω, which was also used to calculate the ω for all anions in this work (data also shown in Figure 6).

### 2.11. Calculation of the effective Born coefficient (ω_e_) for neutral species

The effective Born coefficient used to describe the solvation contribution of neutral species in the revised-HKF model has been calculated from $S_{f}^{0}aq$ [13,22,23] and the Gibbs free energy of hydration (Δ*_hyd_G*^0^) (Appendix C) in the absence of high temperature $C_{P}^{0}$ and *V*^0^ data. It has been demonstrated from empirical data that ω_e_ generally has a negative value for low- molecular weight neutral organic compounds. The negative value is the result of the inflection of $C_{P}^{0}$ and *V*^0^ values towards positive-infinity near the critical point of water. As the properties of a set of molecules become increasingly polar, the solute-solvent interaction increases. In the case of some neutral inorganic polyhydroxyl compounds such as aqueous silica, of which the hydrated form is thought to be H_4_SiO_4_, and boric acid (H_3_BO_3_), both demonstrate electrolyte-like behavior and thus positive ω_e_ values. The mechanism associated with this phenomenon is thought to arise from water-solute versus water-water competition near the critical point [48], where solutes that are associated with more solvent molecules than is the solvent itself, and will have $C_{P}^{0}$ and *V*^0^ values that approach −∞. The volatility of a substance in comparison to water is also thought to have influence over this process as well. This issue is discussed in detail by Amend and Plyasunov [49], where predictions were made concerning the near-critical point behavior of carbohydrates. It is clear, through their discussion and others, that the relationship between *ω_e_* and solvation at higher temperatures is not an obvious one. For instance, the non-electrolyte amino acid proline displays low-temperature solvation behavior that is best described with a negative value for ω_e_. As the temperature increases, however, proline’s solvation is best fit with a positive ω_e_. However, this type of solvation behavior may be particular to the proline zwitterion due to its particularly asymmetrical dipole and spatial arrangement of hydrophobic and hydrophilic moieties [22]. If the high density of polar functional groups, as in polyhydroxy compounds, is to be viewed as an indicator of near-critical behavior of compounds with hydroxyl functionalities, then for dicarboxylic acids, oxalic acid with an ω_e_ that displays neutral behavior may be the closest analogue.

**Figure 7.**
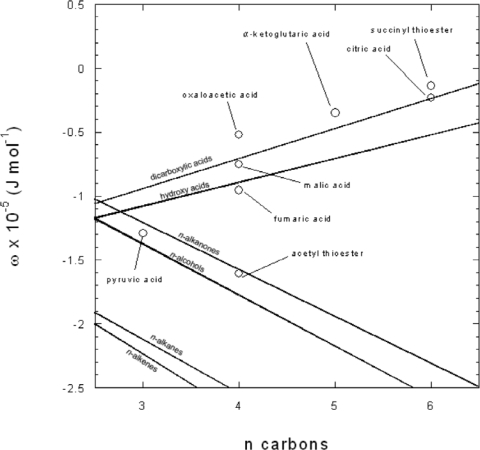
Plot of the effective Born coefficient vs. the number of carbon atoms of neutral organic compounds. The lines are regressions generated from values of the selected functional series of compounds noted in Table 1. Datum points are the values of *ω_e_* for the neutral compounds calculated as described in the text.

In Table 1, different values for *ω_e_* are displayed as calculated from the correlations with the Δ*_hyd_G*^0^ (Equation (44)) and entropy (Equation (45)), as has been done previously. Also included is a systematic estimation of ω_e_ using values provided by Shock and Shock and Helgeson [18,23] with results shown in Figure 7 in comparison to other series of neutral organic compounds.

The values of these estimations were used for ω_e_ in all the neutral compounds in this work. The ω_e_ for pyruvic acid was estimated by taking the ω_e_ from lactic acid and subtracting the difference between *n*-propanol and acetone:

(13) $$\omega_{\text{e}}\;\text{lactic}\;\text{acid}\;-\;(\omega_{\text{e}}\;n-\text{propanol}\;-\;\omega_{\text{e}}\;\text{acetone})=\omega_{\text{e}}\;\text{pyruvic}\;\text{acid}$$

The ω_e_ for oxaloacetic acid was estimated from the value for malonic acid with addition of the carbonyl group value such that:

(14) $$\omega_{\text{e}}\;\text{malonic}\;\text{acid}\;+\;(\omega_{\text{e}}\;2-\text{pentanone}\;-\;\omega_{\text{e}}\;n-\text{butane})=\omega_{\text{e}}\;\text{oxaloacetic}\;\text{acid}$$

Malic acid’s ω_e_ value was estimated by from succinic acid from:

(15) $$\omega_{\text{e}}\;\text{succinic}\;\text{acid}\;+\;(\omega_{\text{e}}\;\text{hydroxybutanoic}\;\text{acid}\;-\;\omega_{\text{e}}\;\text{butanoic}\;\text{acid})=\omega_{\text{e}}\;\text{malic}\;\text{acid}$$

to add the value of the hydroxyl group. For fumaric acid the difference in *n*-alkanes and *n*-alkenes were used to modify succinic acid as:

(16) $$\omega_{\text{e}}\;\text{succinic}\;\text{acid}\;+\;(\omega_{\text{e}}\;n-\text{butene}\;-\;\omega_{\text{e}}\;n-\text{butane})=\omega_{\text{e}}\;\text{fumaric}\;\text{acid}$$

The ω_e_ for α-ketoglutaric acid also used succinic acid and carbonyl value for oxaloacetic acid:

(17) $$\omega_{e}\;\text{succinic}\;\text{acid}\;+\;(\omega_{e}\;2-\text{pentanone}\;-\;\omega_{e}\;n-\text{butane})=\omega_{e}\;\alpha -\text{ketoglutaric}\;\text{acid}$$

Since there are currently no analogous compounds (tricarboxyl) for citric acid, its ω_e_ was estimated to be the same as a C_6_ compound using the slope and y-intercept for dicarboxylic acids. The value for succinyl thioester was estimated by:

(18) $$\omega_{\text{e}}\;\text{hexanoic}\;\text{acid}\;+\;(\omega_{\text{e}}\;2-\text{pentanone}\;-\;\omega_{\text{e}}\;n-\text{butane})\;+(\omega_{\text{e}}\;\text{diethyl}\;\text{sulfide}\;-\omega_{\text{e}}\;n-\text{butane})\;=\;\omega_{\text{e}}\;\text{succinyl}\;\text{thioester}$$

using the value from [22] for diethyl sulfide. The ω_e_ estimation for acetyl thioester also used the same value for diethyl sulfide:

(19) $$\omega_{\text{e}}\;\text{diethyl}\;\text{sulfide}\;+\;(\omega_{\text{e}}\;2-\text{pentanone}\;-\;\omega_{\text{e}}\;n-\text{butane})\;-(\omega_{\text{e}}\;\text{alkane}\;-\omega_{\text{e}}\;{\text{alkane}}_{\left(\text{n}-1\right)})\;=\;\omega_{\text{e}}\;\text{acetyl}\;\text{thioester}.$$

In consideration of consistency with previous works and the pragmatic aspects of the temperature ranges to be expected to be relevant for these compounds, negative values for ω_e_ were chosen. In addition, for practical purposes, since the *p*Ka of the strongest acid among these compounds is 2.49 (pyruvic acid from reference [33]), at pH above 3–4 the acids of these compounds will not usually be germane in writing reactions.

## 3. Results

### 3.1. Analysis of the possible error associated with methods used in estimating reference and standard state parameters

**Figure 8.**
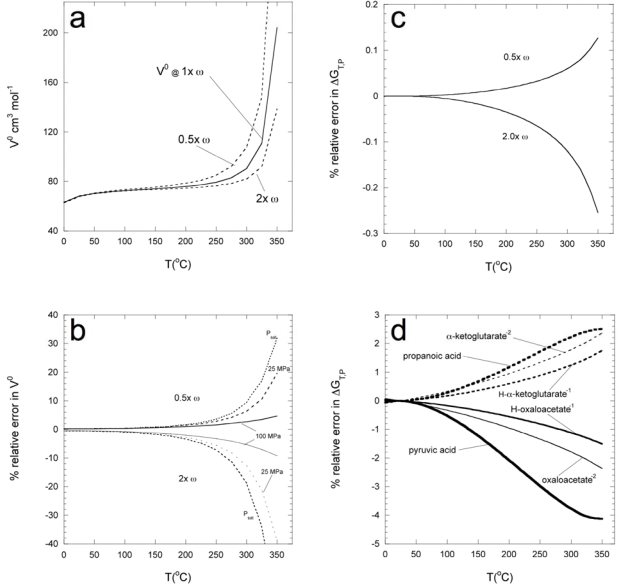
Plot of the error in partial molal properties of neutral and ionic organic compounds expected from the improper estimation of HKF parameters as a function of temperature and pressure. (a) and (b) Partial molal volume of propanoic acid, taken from [23], calculated using Equations ((25) + (26)) with the over- and under-estimation of the effective Born coefficient. (a) The solid line is the predicted *V*^0^ of propanoic acid at Psat using the *ω_e_* from [23]. The upper dashed-line is the *V*^0^ predicted by underestimating *ω_e_* by 0.5-fold. The lower dashed-line is the *V*^0^ predicted by a 2-fold overestimating of *ω_e_*. (b) The percent relative error expected in *V*^0^ as a function of pressure at 0.5x and 2.0x *ω_e_*. (c) The effect of the over- and under-estimation of *ω_e_* on relative error in Δ*G_T,P_* at Psat (from Equations (38) and (39)). (d) Plot demonstrating the relative error in Δ*G_T,P_* at Psat expected from gross misestimation of HKF parameters. Using the reference state $\Delta G_{f}^{0}aq$ (from Table 1) for the labeled acid or ion, the remaining values ( $\Delta S_{f}^{0}aq$, *a_1_*, *a_2_*, *a_3_*, *a_4_*, *c_1_*, *c_2_*, and *ω_e_*) were swapped: propanoic acid (taken from [23] for pyruvic acid, and the respective α-ketoglutarate anion for H-oxaloacetate^−1^ and oxaloacetate^−2^ ions (Table 1), and vice versa.

Since the motivation for this work is to estimate the high temperature and pressure thermochemical parameters for compounds for which there was no data, the credibility of the resulting values need be examined. The effects of errors on the reference state data, as well as for values calculated beyond the reference state, can be analyzed by the comparison of expected calculations, using the estimated values, with calculations from values in which a sensible error is incorporated. Figure 8a demonstrates the influence of the over- and underestimation of *ω_e_* on the partial molal volume of propanoic acid along P_sat_ calculated using Equations (25) and (26) with revised HKF parameters from Shock [23]. Although the reference state $V_{P_{r}T_{r}}^{0}$ is unaffected by ω_e_, the error in the calculated $V_{PT}^{0}$ increases with temperature as the partial molal volume is influenced increasingly by the solvation term, although at higher pressures the error is diminished (Figure 8b). However, as we are most concerned with providing data for reaction thermodynamics at elevated pressure and temperature, the effects of inaccurate estimation of the $\Delta G_{P,T}^{0}$ are of primary concern. As can be seen in Figure 8c, the revised-HKF method is quite insensitive to even a 2-fold under- or over estimation of ω_e_, with a maximum of ~0.3% relative error at 350°C. The insensitivity to errors from the estimation methods can be further tested by swapping the HKF parameters of one compound for another, while using the original $\Delta G_{f}^{0}aq$. To demonstrate this, for compounds in Figure 8d the original $\Delta G_{f}^{0}aq$ values were retained while the remaining values (*S*^0^*aq*, *a_1_*–*a_4_*, *c_1_*, *c_2_*, and Born coefficients) were mutually swapped from another compound: pyruvic acid and propanoic acid, H-oxaloacetate^−1^ and H-α-ketoglutarate^−1^, and oxaloacetate^−2^ and α-ketoglutarate^−2^ (all values are from Table 2 or Shock [23] for propanoic acid) and used to solve Equation (39). The largest error (~4% relative at 350 °C) is encountered when the equation of state parameters from propanoic acid are replaced with those of pyruvic acid. Considering that there is a relative difference (compared to pyruvic acid) of 15%, 24%, and 12% between the *S*^0^*aq*, *V*^0^, and $C_{P}^{0}$ of propanoic acid, respectively, this level of error is quite tolerable, as group additivity estimations generally give relative errors of ~5% for these classes of organic compounds [50]. At 150°C the error is approximately half that at 350°C and is likely to fall below the analytical errors encountered when quantifying the concentrations of these substances.

### 3.2. Estimation of equilibrium constants at high temperatures and pressures

**Figure 9.**
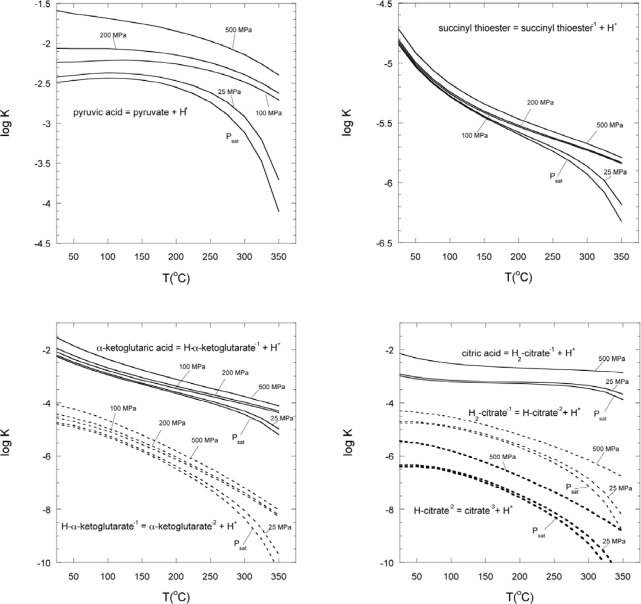
Plot of the logarithms of equilibrium constants for dissociation reactions (as indicated) involving organic species from this work as a function of temperature at P_sat_.

As an example of the usefulness of these data, values of the logarithm of the equilibrium constant (log K) for acid dissociation reactions were calculated at different temperatures from the $\Delta G_{P,T}^{0}$ values at P_sat_ using Equations (38) and (39) and:

(20) $$\text{log}\;K=\frac{-\Delta G^{\circ }}{2.303RT}$$

with the partial molal properties and equation of state parameters in Table 1 (Figure 9). These values allow us to evaluate the potential for each of these reactions to occur under different geochemical conditions. The plots in Figure 8 allow investigation of the pH dependence of the speciation among CAC components. Similarly, the data and parameters, along with the revised HKF equation of state, allow evaluation at wide ranges of temperatures and pressures of reaction energetics among species in the various steps of the CAC (see Figure 1) to determine the thermodynamic viability of these reactions for a variety of conditions. If life did indeed begin under hydrothermal geochemical conditions, these calculations can help identify the conditions necessary for this development.

## 4. Concluding Remarks

Using the thermodynamic data that have either been measured experimentally or estimated through methods described and provided in this paper for the constituents of the citric acid cycle, we can begin to place the fundamental biological process of energy transfer into a geochemical context. With the data and parameters presented in this paper, we can for the first time calculate thermodynamic reaction properties for the citric acid cycle under hydrothermal conditions, whence life may have emerged. Furthermore, we can use calculations such as the ones described above to evaluate the energy cycles of microorganisms that live at elevated temperatures and pressures and thus gain insight into the conditions necessary for the initiation of these cycles. We are now also able to evaluate quantitatively the steps in the reverse or reductive citric acid cycle, which may have preceded the more modern oxidative citric acid cycle as the primary energy transfer mechanism for life. Such calculations are facilitated through use of the computer program SUPCRT92 [51].

In addition, we can gain some insight into the conditions (including factors such as pH, temperature, pressure and concentrations of the chemical components) on the early Earth that may have facilitated the initiation of the central metabolic pathways such as the reductive and oxidative citric acid cycles in biological energy systems by evaluating quantitatively the energy gained through various metabolic reactions.

For example, careful examination of Figure 9 reveals that the logarithms of the equilibrium constants of many of the deprotonation reactions involving components of the citric acid cycle vary by up to an order of magnitude over the known temperature range for life (currently up to 122°C). Because these reactions are functions of pH, changes in the equilibrium constants over the temperature range will change the range of pH at which they would be thermodynamically favorable. A full evaluation of the geochemical parameters attending any environment would be required to determine the effect each would have on reaction favorability and the viability of the citric acid cycle.

## Figures and Tables

**Table 1.** Summary of the aqueous reference state (25 °C, 0.1 MPa) partial molal properties of organic species and parameters for the revised HKF equations of state used to extrapolate to elevated temperatures and pressures.

	**ΔG_f_^0^^a^**	**S^0^^b^**	**V^0^^c^**	**C_P_^0^^b^**	**a_1_^d^,^ee^**	**a_2_^d^**	**a_3_^e^**	**a_4_^f^,^hh^**	**c_1_^g^**	**c_2_^f^**	**ω^b^**

pyruvic acid	−489.1^j^	179.9 k	54.6 h	114.6 h	3.9096	5745.0 ff	2.478	−140079	24.645	384086 jj	−129222 mm
pyruvate^−1^	−474.9 ^I^	171.5 k	41.5 t	−52.8 bb	3.2601	4220.3 ff	17.617	−133776	22.278	−178281 ii	42208 ll
oxalaoacetic acid	−838.3 j	287.5 h	72.4 h	108.7 h	4.8998	7178.9 ff	24.687	−146006	27.207	377592 jj	−52158 mm
H-oxalaoacetate^−1^	−823.7 j	233.1 p	60.3 u	−77.7 cc	4.3045	6672.1 ff	10.473	−143911	−10.600	−181300 ii	330604 ll
oxalaoacetate^−2^	−798.7 ^I^	107.9 q	46.7 v	−328.1 cc	3.8109	5513.3 ff	13.850	−139121	−176.230	−211639 ii	1139579 ll
malic acid	−891.6 k	283.8 h	82.8 w	227.7 w	5.5023	8481.8 ff	21.621	−151393	117.243	509492 jj	−75348 mm
H-malate^−1^	−872.4 l	227.7 r	69.4 x	41.2 dd	4.8234	7890.2 ff	6.924	−148947	106.200	−166880 ii	344473 ll
malate^−2^	−843.1 l	126.8 r	55.7 x	−209.1 dd	4.3174	6702.4 ff	10.385	−144037	−62.817	−197220 ii	1170226 ll
fumaric acid	−645.8 k	261.1 k	78.8 h	154.7 h	5.2807	8963.5 ff	39.867	−153384	58.786	428516 jj	−95650 mm
H-fumarate^−1^	−628.1 k	203.3 k	65.4 y	−31.8 dd	4.6074	7383.1 ff	8.401	−146851	38.350	−175733 ii	373932 ll
fumarate^−2^	−601.9 k	105.4 k	51.7 y	−282.1 dd	4.0998	6191.7 ff	11.873	−141925	−131.082	−206072 ii	1185066 ll
α-ketoglutaric acid	−842.3 j	315.1 h	89.0 z	173.4 h	5.8421	9216.6 ff	24.128	−154430	78.884	449296 jj	−35121 mm
H-α-ketoglutarate^−1^	−829.4 j	243.5 s	75.6 x	−13.1 dd	5.1687	8700.7 ff	4.562	−152298	51.025	−173461 ii	292131 ll
α-ketoglutarate^−2^	−802.0 ^I^	136.0 s	61.9 x	−263.4 dd	4.6661	7520.8 ff	8.000	−147420	−117.057	−203800 ii	1117934 ll
citric acid	−1243.4 m	329.4 l	113.6 w	322.5 w	7.2438	12247.7 ff	39.901	−166961	195.456	614557 jj	−23333 mm
H_2_-citrate^−1^	−1226.3 k	286.2 k	98.1 aa	187.9 k	6.4344	11671.9 ff	−4.096	−164580	241.056	−149109 ii	248464 ll
H-citrate^−2^	−1199.2 k	202.3 k	88.5 aa	0.84 k	6.1522	11009.3 ff	−2.165	−161841	131.407	−171775 ii	1038339 ll
citrate^−3^	−1162.7 k	92.1 k	72.0 aa	−254.8 k	5.4914	9458.3 ff	2.355	−155429	−40.909	−202760 ii	1874470 ll
succinyl thioester	−496.6 n	394.5 h	140.5 h	216.1 h	8.7769	15562.9 ff	115.513	−180666	113.931	496645 jj	−13856 mm
succinyl thioester^−1^	−468.9 o	292.0 h	133.7 h	78.0 h	8.4641	16436.3 ff	−17.979	−184277	133.088	−162424 ii	187278 ll
acetyl thioester	−140.1 n	400.1 h	107.3 h	255.5 h	6.9314	10305.7 gg	36.887	−158933	171.102	342400 kk	−160625 mm

^a^-kJ mol^−1^.

^b^- J mol^−1^ K^−1^.

^c^-cm^3^ mol^−1^.

^d^-J mol^−1^ bar^−1^.

^e^- J mol^−1^.

^f^-J K mol^−1^.

^g^- J mol^−1^ K^−1^.

^h^-value as described in Table 1.

^i^- from [33].

^j^-calculated from *p*Ka values from [33] and $\Delta G_{f}^{0}aq$ of oxaloacetate^−2^ from above.

^k^-from [38].

^l^-from [37].

^m^-from [41].

^n^-estimated as described in Figure 2.

^o^-estimated as was succinyl thioester in Figure 2, except using pentanoate, taken from [23], as the base structure.

^p^- estimated as was oxaloacetic acid in Figure 2, except using H-malonate^−1^, taken from [23], as the base structure.

^q^- estimated as was oxaloacetic acid in Figure 2, except using malonate^−2^, taken from [23], as the base structure.

^r^-calculated from $S_{f}^{0}aq$ of malic acid in above table using the Δ*_ion_S* from [33].

^s^-estimated from $S_{f}^{0}aq$ of *a*-ketoglutaric acid from above table assuming the same Δ*_ion_S* as between succinic acid and its respective ions in [23].

^t^-estimated from *V*^0^ of pyruvic acid in above table assuming the same Δ*_ion_V* as between lactic acid and lactate, in [23].

^u^-estimated as was oxaloacetic acid in Figure 2, but using H-malonate^−1^, from [23] as the base structure.

^v^- estimated as was oxaloacetic acid in Figure 2, but using malonate^−2^, from [23] as the base structure.

^w^-from [42].

^x^-estimated by assuming the same Δ*_ion_V* as between succinic acid, taken from [35] and its respective ions, taken from [23].

^y^-from [43].

^z^-from [36].

^aa^-from [44].

^bb^-estimated from $C_{P}^{0}$ of pyruvic acid in above table assuming the same $\Delta_{ion}C_{P}^{0}$ as between lactic acid and lactate in [23].

^cc^-estimated as described in Figure 2 for oxaloacetic acid but using respective $C_{P}^{0}$ of H-malonate^−1^ or malonate^−2^.

^dd^-estimated form the acid in the above table assuming the same $\Delta_{ion}C_{P}^{0}$ as between succinic acid and the respective −1 or −2 ion from [23].

^ee^ – estimated using Equation (6)

^ff^-estimated using Equation (7).

^gg^- estimated using Equation (8).

^hh^-calculated from the *a_2_* parameter in above table as described by [23].

^ii^- calculated using Equation (10).

^jj^- calculated using Equation (11).

^kk^- calculated using Equation (12).

^ll^-calculated from $S_{f}^{0}aq$ in above table using Equations (30–32) from [18].

^mm^-estimated as described in text.

**Table 2.** The effective Born functions (ω J mol^−1^) used for neutral organic species calculated by different methods.

	**ω^a^**	**ω^b^**	**ω^c^**
pyruvic acid	−1.2922	−1.2679	1.2156
oxalaoacetic acid	−0.5216	−0.4495	2.4908
malic acid	−0.7535	−0.5100	2.7785
fumaric acid	−0.9565	−0.7306	−1.7418
a-ketoglutaric acid	−0.3512	−0.2814	1.3690
citric acid	−0.2333	−0.2780	4.0067
succinnyl thioester	−0.1386	0.0107	7.7939
acetyl thioester	−1.6063	0.0453	−3.7706

^a^-calculated as described in text and Figure 6.

^b^-calculated from aqueous entropies from Table 4 using Equation (45).

^c^-calculated from the hydration Gibbs free energy using Equation (44).

## Appendices

### Appendix A

The *S*^0^*aq* of ethyl sulfide was calculated from Equation (21) using values of $\Delta S_{f}^{0}l$ [29], Δ*_hyd_H*^0^, Δ*_hyd_G*^0^ [52], and Δ*_vap_H*^0^ [53], along with:

(21) $$S_{aq}^{0}=\frac{\Delta_{hyd}H^{0}-\Delta_{hyd}G^{0}}{T}+\frac{\Delta_{vap}H^{0}-\Delta_{vap}G^{0}}{T}+S_{l,s}^{0}$$

and the $\Delta G_{f}^{0}g$ [52] and $\Delta G_{f}^{0}l$ [28], which were used to calculate Δ*_vap_G*^0^ from the relationship:

(22) $$\Delta_{vap}G^{0}=\Delta G_{f}^{0}g\;-\;\Delta G_{f}^{0}l\;\;.$$

### Appendix B

The revised HKF eos combines substance specific structural or non-solvation ( $\Delta \;\mu_{n}^{0}$) and solvent dependent or solvation- $\Delta \;\mu_{n}^{0}$ partial molal properties to predict the conventional standard molal properties(*μ*^0^) of aqueous species:

(23) $$\mu^{0}=\Delta \mu_{n}^{0}+\Delta \mu_{s}^{0}$$

The non-solvation contribution to the standard molal term is a summation of the intrinsic property of a substance and its affect the solvent structure in its local vicinity and is the dominant contribution to the partial molal quantity at temperatures of ~≤ 150 °C. The solvent contribution to the standard molal term is a factor of the intrinsic properties of water and the Born transfer properties of solvation [11]. In effect, for a given structural moiety of a compound the greater the degree or hydrophilicity (i.e. compounds that form hydrogen bonds and/or ions), will reflect in the collapse of the local solvent structure, whereas more hydrophobic moieties will necessitate the formation of cavities within the solvent. The solvation contribution to a standard molal property has the greatest affect when the solvent (H_2_O) is undergoing the greatest degree of change in permitivity, volume, and heat capacity as these functions are influenced by high temperature and pressure. The non-solvation and solvation contribution to the partial molal volume for ions, electrolytes, and neutral aqueous organic species is defined by:

(24) $$V^{0}=\Delta V_{n}^{0}+\Delta V_{s}^{0}$$

where the non-solvation term is defined by:

(25) $$\Delta V_{n}^{0}=a_{1}+\frac{a_{2}}{\Psi \;+\;P}+\left(a_{3}+\frac{a_{4}}{\Psi \;+\;P}\right)\left(\frac{1}{T-\Theta }\right)$$

and the solvation term for ions and electrolytes is:

(26) $$\Delta V_{s}^{0}=-\omega Q+\left(\frac{1}{ɛ}-1\right){\left(\frac{\partial \omega }{\partial P}\right)}_{T}$$

For neutral organic species, the non-solvation term partial derivative function (*∂ω*/*∂P*)*_T_* is taken to be zero, simplifying the term to −ω_e_Q since the effective Born coefficient is used.

The combination of non-solvation and solvation function for integration of the heat capacity function is:

(27) $$C_{P}^{0}=\Delta C_{P,n}^{0}+\Delta C_{P,s}^{0}$$

where the non-solvation contribution is defined by:

(28) $$\Delta C_{P,n}^{0}=c_{1}+\frac{c_{2}}{{\left(T-\Theta \right)}^{2}}-\left(\frac{2T}{{\left(T-\Theta \right)}^{3}}\right)\left[a_{3}(P-P_{r})+a_{4}\;\text{ln}\left(\frac{\left(\Psi \;+\;P\right)}{\left(\Psi \;+\;P_{r}\right)}\right)\right]$$

and the solvation term for ions and electrolytes is:

(29) $$\Delta C_{P,s}^{0}=\omega TX+2TY{\left(\frac{\partial \omega }{\partial T}\right)}_{P}-T\left(\frac{1}{ɛ}-1\right){\left(\frac{\partial^{2}\omega }{\partial T^{2}}\right)}_{P}$$

and reduces to ω_e_TX for neutral organic species, where (*∂ω*/*∂T*)*_P_* and (*∂*^2^*ω/∂P*^2^)*_P_* terms become zero. The *a*_1_, *a*_2_, *a*_3_, *a*_4_, *c*_1_, and *c*_2_ coefficients define the substance-specific non-solvation parameters and ω and ω_e_ parameters are the conventional and effective Born coefficients for ionic species or electrolytes and neutral species, respectively. The Q, X, and Y coefficients are the solvent, and P-T dependent Born functions from [11]. Ψ and Θ are the solvent parameters corresponding to 2600 bar and 228 K, respectively. T and P are the temperature and pressure of interest, respectively, and the T_r_ and P_r_ terms are those of the reference state temperature of 298.15 K and pressure of 0.1 MPa.

The conventional Born coefficient of an ion was calculated by first utilizing the correlation of the standard partial molal entropy with the effective electrostatic radius (r_e_) of the *jth* aqueous species (from [17]) at 1 bar and 25 °C, such that:

(30) $$r_{e,j}=\frac{Z_{j}^{2}(\eta Y-100)}{S_{j}^{0}aq-\alpha_{z}}$$

where Z*_j_* is the charge of the j th aqueous species, η is the quantity equal to N^0^*e*^2^ / 2 (N^0^ is Avogadro’s number 6.02252 × 10^23^ mol ^−1^, and e is the absolute electronic charge (*esu*) of 4.80898× 10^−10^). Y is the Born function at 25^0^ C and 1 bar [47] and α_z_ is the charge-dependent factor from [17], equal to 72, 141, and 211 for mono-, di-, and trivalent anions, respectively. The effective electrostatic radius is the related to the conventional Born coefficient of the *j* th ionic species (ω*_j_*) by:

(31) $$\omega_{j}\;=\;\omega_{j}^{abs}\;-\;Z_{j}\;\omega_{H^{+}}^{abs}$$

where:

(32) $$\omega_{j}^{abs}=\frac{N^{0}e^{2}Z_{j}^{2}}{2r_{e,j}}=\frac{\eta Z_{j}^{2}}{r_{e,j}}$$

The factor $\omega_{H^{+}}^{abs}$ is the absolute Born coefficent for the hydrogen ion equal to 0.5387 × 10^5^ cal mol^−1^ at 1 bar and 25 °C [54]. The partial and second derivative functions for the conventional Born coefficient for ionic species at T and P were calculated with Equations (33–35) from [47] using *X*_1_ and *X*_2_ values using Equations (36) and (37) (from [11]), respectively.

(33) $${\left(\frac{\partial \omega }{\partial P}\right)}_{T}=X_{1}{\left(\frac{\partial g}{\partial P}\right)}_{T}$$

(34) $${\left(\frac{\partial^{2}\omega }{\partial T^{2}}\right)}_{P}=X_{2}{\left(\frac{\partial g}{\partial T}\right)}_{P}^{2}+X_{1}{\left(\frac{\partial^{2}g}{\partial T^{2}}\right)}_{P}$$

(35) $${\left(\frac{\partial^{2}\omega }{\partial P^{2}}\right)}_{T}=X_{2}{\left(\frac{\partial g}{\partial P}\right)}_{T}^{2}+X_{1}{\left(\frac{\partial^{2}g}{\partial P^{2}}\right)}_{T}$$

(36) $$X_{1}\equiv -\eta \sum_{j=1}^{\overset{⌢}{j}}k_{j}[Z_{j}^{3}|r_{e,j}^{-2}-Z_{j}\;{\left(3.082\;+\;g\right)}^{-2}]$$

(37) $$X_{2}\equiv 2\eta \sum_{j=1}^{\overset{⌢}{j}}k_{j}\left[Z_{j}^{4}|r_{e,j}^{-3}-Z_{j}\;{\left(3.082\;+\;g\right)}^{-3}\right]$$

The variable η corresponds to the quantity equal to N^0^*e*^2^ / 2 (N^0^ is Avogadro’s number 6.02252 × 10^23^ mol ^−1^, and e is the absolute electronic charge (*esu*) of 4.80898× 10^−10^), *k* and *Z* are the kth electrolyte and charge of the its *j* th species, respectively (from [11]). The term g is the solvent dependent function from [47].

(38) $$\Delta G^{0}\equiv \Delta G_{f}^{0}+\left(\Delta G_{P,T}^{0}-\Delta G_{\text{Pr},Tr}^{0}\right)$$

(39) $$\begin{array}{l} \Delta G_{P,T}^{0}-\Delta G_{\text{Pr},Tr}^{0}=\\ -\Delta S_{\text{Pr},Tr}^{0}(T-T_{r})-c_{1}\left(T\;\text{ln}\left(\frac{T}{T_{r}}\right)-T+T_{r}\right)+a_{1}(P-P_{r})+a_{2}\text{ln}\left(\frac{\Psi +P}{\Psi +P_{r}}\right)\\ -c_{2}\left\{\left[\left(\frac{1}{T-\Theta }\right)-\left(\frac{1}{T_{r}-\Theta }\right)\right]\left(\frac{\Theta -T}{\Theta }\right)-\frac{T}{\Theta^{2}}\text{ln}\left(\frac{T_{r}(T-\Theta )}{T(T_{r}-\Theta )}\right)\right\}\\ +\left(\frac{1}{T-\Theta }\right)\left[a_{3}(P-P_{r})+a_{4}\;\text{ln}\left(\frac{\Psi +P}{\Psi +P_{r}}\right)\right]\\ +\omega \left(\frac{1}{{ɛ}_{P,T}}-1\right)-\omega \left(\frac{1}{{ɛ}_{P_{r},T_{r}}}-1\right)+\omega Y_{P_{r},T_{r}}(T-T_{r}) \end{array}$$

### Appendix C

#### Calculation of the effective Born coefficient (w_e_) from Δ_hyd_G^0^

The effective Born coefficient (ω_e_) for neutral organics was calculated form the correlation described by [50] with the Gibbs free energy of hydration (Δ*_hyd_G*^0^) and the Henry’s constant of a neutral species. Since the Henry’s constant (*K*_H_) approximates the equilibrium constant [50] for a reaction:

(40) $$A_{g}=A_{aq}$$

the *K*_H_ is:

(41) $$K_{H}=\frac{C_{aq}}{C_{g}}$$

where C_aq_ is the moles of solute in solution and C_g_ is the equilibrium vapor pressure (MPa) of the solute. If both fugacity and molality are considered to be unity for the gas and aqueous species, repectively, then the Δ*_hyd_G*^0^ can be calculated by:

(42) $$\Delta_{hyd}G^{0}=-RT\;\text{ln}K_{H}$$

Henry’s constant values for pyruvic acid [55], citric, malic, and α-ketoglutaric acids [56], and acetyl thioester [52] were used. The Δ*_hyd_G*^0^ for oxaloacetic and fumaric acids, and succinyl thioester were calculated from the $\Delta G_{f}^{0}aq$ from Table 3 and the $\Delta G_{f}^{0}g$ calculated from [28] by:

(43) $$\Delta_{hyd}G^{0}=\;\Delta G_{f}^{0}aq\;-\;G_{f}^{0}g$$

Then using the correlation from [50]:

(44) $$\omega_{e}\times \;10^{\;5}=2.61+\frac{324\;.1}{\Delta {\;}_{hyd}G^{\;0}-90\;.6}$$

#### Calculation of the effective Born coefficient (w_e_) from $S_{f}^{0}aq$

Aqueous entropy values have been used to estimate the effective Born coefficient for different classes of neutral organic compounds [18,22,23]. The correlation used for the neutral compounds in this study were determined by Shock [23]:

(45) $$\omega_{e}\;\times \;10^{5}\;=\;S_{f}^{0}\;\times \;2770\;-245900$$

where all values are in J·mol^−1^.
